# Speech Facilitates the Categorization of Motions in 9-Month-Old Infants

**DOI:** 10.3389/fpsyg.2018.02146

**Published:** 2018-11-20

**Authors:** Micah B. Goldwater, R. Jason Brunt, Catherine H. Echols

**Affiliations:** ^1^School of Psychology, University of Sydney, Sydney, NSW, Australia; ^2^Rosemead School of Psychology, Biola University, La Mirada, CA, United States; ^3^Department of Psychology, University of Texas at Austin, Austin, TX, United States

**Keywords:** infancy, categorization, habituation, motion perception, category learning, language and thought

## Abstract

Two experiments were used to investigate the influence of both native and non-native speech on the categorization of a set of an object’s motions by 9-month-olds. In Experiment 1, infants were habituated to a set of three object motions and tested with familiar and novel motions. Results of Experiment 1 show that infants were more likely to categorize the motion stimuli if they listened to either the native or non-native speech during the categorization process than if they listened to music or heard nothing at all. Results of Experiment 2 show that discrimination of the motions was not impaired by the presence of the labeling phrases. These results are consistent with a number of findings that report a unique influence of labels on categorization of static objects in infancy and extend those findings to categorization of motions.

## Introduction

Before infants are old enough to produce labels or even comprehend that labels refer to categories, the presence of human speech may have an effect on infants’ processing of visual stimuli (e.g., Roberts and Jacob, 1991; Balaban and Waxman, 1997; Nazzi and Gopnik, 2001; Fulkerson and Haaf, 2003; Robinson and Sloutsky, 2007; Plunkett et al., 2008). These findings raise the intriguing possibility that, from early in development, language may help to shape our basic categories. However, caution is required, given several questions are left open by this research. A first question concerns the nature of the effects obtained in this research – whether they truly reflect categorization as opposed to other types of effects on visual processing. A second question is whether it is speech that is responsible for these effects as opposed to any complex acoustic stimuli. A third question concerns the kinds of categorization that speech might promote, whether only object categorization is facilitated or whether speech can influence categorization of a broad range of stimuli – possibly any type of regularity present in the visual input. Addressing these questions will provide insights into the ways in which language might influence the fundamental ability of humans to organize their world through categorization

To help answer each of these questions, we refer to the definition of categorization offered by Younger and Cohen (1983) – categorization is the treatment of a discriminable set of stimuli as an equivalence class. This definition entails that categorization is measured by both the ability to generalize between exemplars within a category, and the ability to recognize that distinct exemplars are in fact distinct exemplars.

This definition is important to understand the debate around the first question concerning the nature of language’s effects. One hypothesis is that words facilitate categorization of visual stimuli, having a direct effect on the categorical structure of information in working memory. Evidence has been presented that labels can enable categorization of stimuli that are otherwise not categorized in silence (Fulkerson and Haaf, 2003), and that labels may prompt different category formations of stimuli that are otherwise categorized in silence (Plunkett et al., 2008). Alternatively, it may be the case that what appears to be evidence of speech facilitating or influencing categorization actually reflects a reduction in discrimination of in-category exemplars (Robinson and Sloutsky, 2004, 2007).

A related question is how specifically speech-related these effects on visual processing might be. If there is an effect of speech on visual processing, that effect might be unique to speech (Balaban and Waxman, 1997), or related more generally across a range of acoustic stimuli (Roberts and Jacob, 1991). It might also be that experience with acoustic stimuli, or classes of acoustic stimuli, contributes to the range of acoustic stimuli across which any effect generalizes (Robinson and Sloutsky, 2007; Sloutsky and Robinson, 2008).

A final issue is the type of categorization that speech affects. Most previous studies focus on object categories. However, humans categorize motions, spatial relations, social roles, properties, and so forth, and it is unclear how speech effects extend to these other kinds of categories (e.g., see Gentner, 1982; Markman and Stilwell, 2001 for discussion). In this paper, we examine whether speech uniquely facilitates categorization of motions without reducing discrimination of within category exemplars.

### What Is the Effect of Speech on Visual Categorization? Contrasting Methods and Measures for Categorization and Discrimination

The primary question of interest here is whether language has some effect on visual processing. As mentioned above, one hypothesis is that hearing speech facilitates categorization of visual stimuli. Balaban and Waxman (1997) were first to show that labels affect categorization in infants as young as 9 months. In their study, infants were familiarized to drawings of a familiar animal category (e.g., pigs). At test, they were shown a novel exemplar from the familiarized category, and a novel out-of-category (OOC) exemplar (e.g., a cow). Categorization was measured by a novelty preference for the novel OOC relative to the novel in-category exemplar. The reasoning is that, although both images are novel, categorization would lead to this new pig being treated as relatively familiar because it is another instance of a familiar category. There was a preference for OOC stimuli, and it was larger for participants who heard a label during familiarization than for participants who heard a tone. Waxman and her colleagues have published several papers on this topic in the twenty years since, replicating the effect of speech using the same basic task structure and measures (e.g., Ferry et al., 2010).

Plunkett et al. (2008) familiarized 10-month-old infants to a set of novel animals that could be either categorized into two sub categories or one larger category. Categorizing the set into one or two groups during familiarization changed which of two novel stimuli at test were treated as relatively more novel (i.e., which was seen as outside the category-ies). Infants treated the set as containing two categories when they viewed the set in silence. Infants treated the same set of stimuli as containing one category when they viewed the set in the presence of a single label. These findings show that, even if infants can form categories in silence, labels can influence the specific structure of the categories.

An alternative explanation for some of these findings has been advanced by Robinson and Sloutsky (2004, 2007) who argue that acoustic stimuli such as labels demand attentional resources that would otherwise be used for visual processing, leading to poorer visual discrimination. Robinson and Sloutsky describe this interference as overshadowing, and have further posited different effects depending on the level of overshadowing that occurs. If overshadowing is strong enough, then infants should fail to distinguish between visual stimuli, even across categories. If this strong overshadowing effect were to occur in a study comparing looking times to a novel in-category stimulus and a novel OOC stimulus, then infants’ looking times for the two stimuli would be similar, leading to a conclusion that there was no categorization.

Robinson and Sloutsky posit a different outcome for a moderate level of overshadowing. If an acoustic stimulus overshadows visual processing only moderately, they argue, infants may fail to encode the finer, in-category variation between stimuli, but still encode the gross differences between exemplars from different categories (Robinson and Sloutsky, 2004). An inability to discriminate in-category contrasts after exposure to a set of category members would lead to a lack of preference for a novel in-category exemplar relative to familiar in-category stimuli. The ability to discriminate across category contrasts would lead to a preference for a member of a novel category. If stimulus discrimination is not tested, this situation might incorrectly be interpreted as successful categorization, rather than an inability to discriminate.

Robinson and Sloutsky (2007) present results of a categorization study with 8- and 12-months-olds in which the infants are familiarized to a natural category (cats). They then use the same kind of comparison as Balaban and Waxman (1997) at test, and measure categorization as a preference for a novel OOC stimulus over a novel in-category stimulus (relative to baseline measures of preference for the novel stimuli). The infants show this OOC preference in silence, but not in the presence of labels. This is interpreted to mean that the presence of labels reduced the level of children’s visual processing, and as a result, categorization, thus supporting the overshadowing hypothesis.

Relative to our research question, one drawback of the designs by Plunkett et al. (2008) and Robinson and Sloutsky (2007) is that they do not test a stimulus set that infants fail to categorize in the absence of labeling. Our research question is whether labeling speech can facilitate categorization without reducing discrimination. To test if it can facilitate categorization, one needs to test stimuli that participants do not categorize in silence. Also, the results are stronger if the category is artificial, as it is in Plunkett et al., but not in Balaban and Waxman or Robinson and Sloutsky (c.f., Robinson and Sloutsky, 2010). Artificial stimuli strengthen the claim that the speech enabled category learning, and that it did not simply cue a known category (Plunkett et al., 2008). We present experiments that test for the facilitation of categorization by speech using an artificial category that infants do not learn in silence.

Again, our working definition is that categorization is the treatment of a set of discriminable stimuli as an equivalence class (Younger and Cohen, 1983). The most straightforward way to operationalize this definition is by showing that there is generalization from familiar in-category stimuli to novel in-category stimuli. In terms of infant studies, after an initial familiarization or habituation phase, this would be shown by a lack of novelty preference for a novel in-category stimulus when compared to a familiar stimulus. That is, categorization is demonstrated during a post-habituation test phase when an exemplar from within the category *not shown* during the habituation phase elicits an equally low looking time as an exemplar from the category *shown* during the habituation. This lack of novelty preference means that infants are generalizing their habituation from one to the other – that novel and familiar exemplars from the same category form an equivalence class.

The measure used in the studies reviewed above is somewhat different. After a familiarization or habituation phase, those studies compare a novel in-category stimulus to a novel OOC stimulus, and argue categorization is shown when there is a novelty preference (i.e., longer looking times) to the OOC stimulus. Although that measure provides interesting information, and could reflect categorization, it is not the strictest or most direct measure of categorization. The infant does not need to form an equivalence class containing the novel and familiar in-category stimuli, that is generalize across them, to show a novelty preference for the OOC stimulus. To show a novelty preference (i.e., look longer) to the OOC stimulus, it is sufficient to represent the stimuli along a similarity gradient and recognize that the OOC stimulus is less similar to the familiarized stimuli than the novel in-category is.

We argue that the lack of a novelty preference between novel and familiar in-category stimuli is the measure of categorization that derives most closely from the definition of categorization. However there are two potential disadvantages to this approach, but we address each below: (1) It requires an independent test of discrimination, because a lack of discrimination could also explain the lack of novelty preference. (2) This measure is a null effect, that is, there is an equally low looking time for familiar and novel category members.

Cohen and Strauss (1979) satisfied the concern about the lack of discrimination by establishing the “generalization of habituation” paradigm. This study had three habituation conditions. In the first, the infants habituated to a single face at a single angle. In the second, infants habituated to a single face at multiple angles. In the third, infants habituated to multiple faces. In the first condition, infants showed a novelty preference to the familiar face at a new angle. In the second condition, infants generalized their habituation, that is, did not show a novelty preference to the familiar face at a new angle, but did show a novelty preference to a new face. In the third, habituation was generalized to a novel face. Infants became habituated to the *regularity* in the stimuli, not the individual stimuli. Although the second and third conditions showed a lack of novelty preference to novel stimuli, one can be sure infants could discriminate because of the results of the first condition.

Our experiments use this logic to argue for categorization and test for reduced discrimination. After initially testing the stimuli for discriminability (see Methods below), we conducted an experiment that habituated infants to three exemplars of an artificial motion category. We then tested them with one of the familiar exemplars and a novel within category exemplar to determine whether they were habituated to the exemplars as a category or to the exemplars as unrelated individuals. As mentioned above, there were four audio conditions (silence, English speech, Hebrew speech, and didgeridoo music). Novelty preference at test is evidence for discrimination between habituation and test stimuli. However, the absence of a novelty preference at test could result from generalization to the test stimulus (i.e., the novel within-category exemplar) or from a lack of discrimination between the habituation and test stimuli. So, we also conducted a follow-up discrimination study, habituating infants to just a single exemplar, with the audio conditions that showed generalization of habituation in the first study.

The second issue concerns the null effect more generally. If looking time is equally low to two key test trials (familiar and novel within-category exemplars), it may be that infants are incapable of showing a novelty preference at this point in processing (Hunter and Ames, 1988). We test for such a possibility by presenting infants with an OOC stimulus. Using an OOC trial also gives us a chance to test directly whether the novel, in-category stimuli will be treated like a familiar stimulus, or will be treated like the OOC stimulus, showing categorization or lack there-of, respectively.

### Are These Effects Unique to Speech?

Here, as with the first issue, there are also conflicting findings. Balaban and Waxman (1997) show that a tone does not produce the same facilitatory effect that speech does. Contrary to these results and conclusions, work reported by Roberts and Jacob (1991) suggests that the facilitative effect of language on categorization might not be unique, even at 15 months (see also Campbell and Namy, 2003). Roberts and Jacob show that presenting either music or speech will increase the likelihood of visual categorization by 14- to 15-month-old infants. The seemingly contradictory findings might be explainable by methodological differences between the studies reported by Roberts and Jacob and those by others. Roberts and Jacob used a habituation paradigm instead of the familiarization procedure used by Balaban and Waxman (1997). This may have allowed for sufficient time for the infants in Roberts and Jacobs’ non-language sound condition to process the stimuli as a category. Roberts and Jacob also used a more complex acoustic stimulus than did Balaban and Waxman (music as opposed to a sine wave tone). The greater complexity of the music stimuli may have attracted infants’ attention to a greater degree than the simple tone (though complicating the matter, non-human primate vocalizations facilitate infants’ categorization when they are 4 months old, but not when they are 5 months old; Ferry et al., 2013). These differences separately or together may have contributed to the likelihood that infants categorized the visual stimuli in the non-language conditions. These contradictory findings suggest, however, that the issue of whether language is unique in promoting categorization is not fully resolved. We will add to this literature by using complex voice-like music made using an Australian Aboriginal instrument, the didgeridoo.

In addition to the music, we make another contribution to this area of study with the use of non-native speech. We use Hebrew, which has quite a different sound system from English. Without similar previous work, it is unclear what predictions to make, that is, to what degree the effect of speech might rely on familiar speech sounds. On the one hand, children of this age (9 months old) can discriminate native from non-native languages based on prosody (Mehler et al., 1988), as well as sound sequences and individual speech sounds (Jusczyk et al., 1993). On the other hand, there is also a larger overlap of acoustic variation shared by human produced language compared to the differences across non-human and non-language classes of acoustic stimuli, and it is often not until between 10 and 12 months that infants tend to stop showing discrimination for phonetic contrasts not present in their native language (e.g., see Best, 1993). We therefore predict that the effects of language at 9 months of age will not be specific to any single language, but are rather effects of human voice producing language.

With this issue of the uniqueness to speech, the overshadowing hypothesis makes interesting predictions. Robinson and Sloutsky (2007) included a condition in which infants were presented with an unfamiliar sound. Twelve-month-old infants in this condition were even less likely than in the language condition to prefer to look toward the OOC exemplar over the in-category exemplar during test. Robinson and Sloutsky argue that labels, as a familiar class of stimuli, may demand less attentional resources, and therefore, the “overshadowing” effect should be less in label conditions than in conditions of unfamiliar noise. They use this pattern to reinterpret the label over tone advantage in Balaban and Waxman (1997) not as an advantage of categorization, but as a smaller disadvantage for labels relative to tones, that is, it was due to a lesser degree of overshadowing.

According to the auditory overshadowing hypothesis, the infants in our silent condition should be able to show a novelty preference at test to the new category member, but they should be less and less likely to show this same novelty preference as stimuli decreases in familiarity, from English speech to Hebrew speech to the didgeridoo music. However, our account, along with Balaban and Waxman (1997) (see also Fulkerson and Haaf, 2003; Fulkerson and Waxman, 2007; Ferry et al., 2010), predicts that didgeridoo music and silence should pattern together, and differently from the two speech conditions.

### What Kind of Categories Do Labels Affect?

The third issue addressed in this paper concerns the nature of the category to be formed. Is the relationship between labels and object categories somehow privileged over other types of categories, as suggested by some well-known approaches to word learning (Markman, 1990; Waxman, 2003)? These influential approaches propose that the relationship between labels and objects (or object categories) is somehow special (Markman, 1990), or that assigning labels to object categories is less complex than to categories of motions that dynamically change the relations between objects (Gentner, 1982).

An object category bias, though, is not the only possibility. Another possibility is that labels are associated with aspects of experience that are consistent across repetitions of a label. Consistent with this view, Oviatt (1980) posited a precursor to true word learning that she termed “recognitory comprehension” in which a label is recognized as an associate of some regular set of information in the environment. There has also been a suggestion that the presence of consistent labels may help children to form categories of spatial relationships, in much the same way as has been discussed above for object categorization (Bowerman and Choi, 2001). In this early stage of language development, all labels may be equally well able to become associated with any statistical regularity in the environment, while later in development, as children learn about the relevant grammatical categories provided by language, some types of labels become associated with object categories (common nouns), others with properties (adjectives), and others associated with actions (verbs) (Waxman, 1999; Waxman and Booth, 2000; Echols and Marti, 2004).

There has been some empirical support for this theoretical point of view. Researchers have discovered that, as with objects (Schafer and Plunkett, 1998; Werker et al., 1998), infants are able to associate labels with actions in a laboratory setting. Casasola and Cohen (2000), using a habituation-switch paradigm, found that 18-month-olds, but not 14-month-olds were able to form associations between labels and causal actions such as pushing and pulling. Casasola and Wilbourn (2004) showed that 14-month-olds were able to associate novel labels with the actions of “placing on” and “placing off.” These findings that infants associate object categories with labels (Werker et al., 1998) at roughly the same age that they associate other kinds of categories with labels leads directly to question at hand: will potential precursors to label learning, such as the facilitation of categorization, also exist for motion categories?

### Summary of Predictions

**FIGURE 1:**
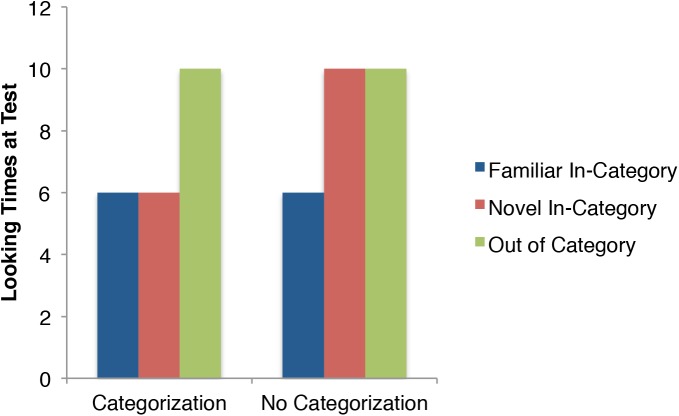
Idealized looking times of the three critical test trials showing patterns of categorization, and a lack of categorization.

We present two experiments investigating the effects of auditory stimuli (English and Hebrew speech, didgeridoo music, and silence) on the categorization and discrimination of a novel motion category. Figure 1 shows idealized versions of looking times reflecting categorization and the lack thereof across the three critical test trials: familiar in-category; novel in-category, and novel OOC. Regardless of whether the infants categorize the stimuli, they should look briefly at the familiar exemplar, and look for much longer at the OOC exemplar. Even without recognizing that each individual stimulus during habituation was common to a shared category, the OOC stimulus is strikingly dissimilar to every stimulus they saw up to that point, and thus infants should find this stimulus surprising. The critical trial is the novel in-category exemplar: if the infants categorized the stimuli during habituation, they will generalize their habituation to this novel exemplar and show looking times quite similar to the familiar exemplar. If, however, they have not categorized the stimuli, this trial should have longer looking times, more closely resembling the OOC exemplar.

We predicted that for Experiment 1, labeling speech, regardless of language, would facilitate categorization, while infants would not categorize the stimuli in silence or while hearing the didgeridoo music. This pattern would be revealed by an interaction of condition (speech vs. non-speech) and test trial. Further, if speech truly supports categorization and is not actually reducing the ability of the infants to discriminate exemplars from the same category, then in Experiment 2, when infants only habituate to a single exemplar while exposed to speech, they should now show the lack of categorization pattern, when both novel in- and OOC exemplars elicit long looking times.

## Experiment 1

### Methods

This study was approved by the Institutional Review Board of the University of Texas at Austin. We obtained written informed consent from the participants’ parents or legal guardians in line with the study approval.

#### Participants

One hundred eighteen 9-month old (±2 weeks; *M* = 274 days; *SD* = 9.1 days) infants participated (72 boys and 62 girls). We chose 9 month olds to be consistent with Balaban and Waxman (1997), and it is an age at which we can be confident that infants can discriminate between native and non-native languages (e.g., Jusczyk et al., 1993). We only recruited infants who primarily heard English in their homes, and none that were exposed to Hebrew on any sort of regular basis. The participants were recruited by letter and phone using a database of potential participants maintained at a university research lab. Participants were given a t-shirt in appreciation of their participation.

Three infants were excluded due to computer error; and five were excluded because there was no sign of habituation, having attended 20 consecutive trials without reaching criterion. Four infants were excluded for maximum looking times on the first test trial. This left 106 infants contributing to the data analysis.

#### Stimuli

**FIGURE 2:**
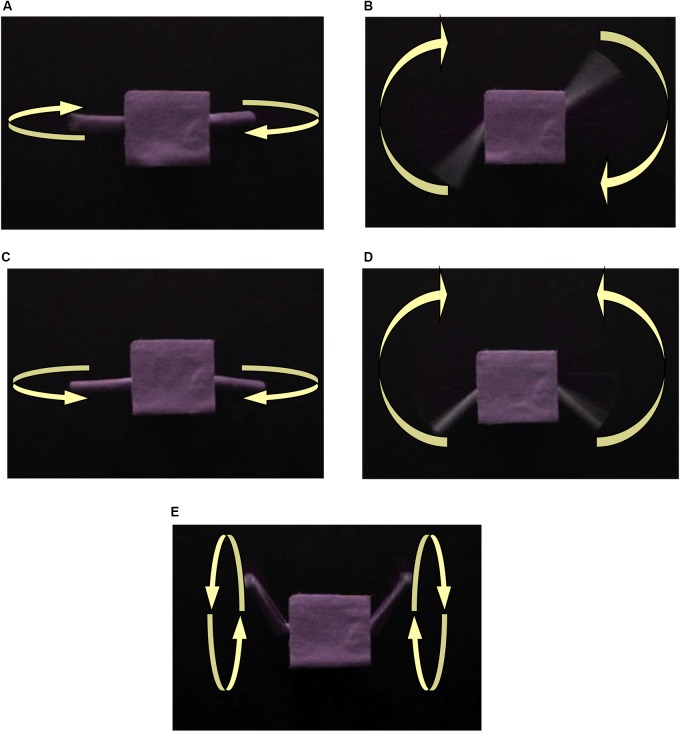
Motions A, B, C, and D are a category of motions. Motion E is the out-of-category movement. The arms of the object move in the following manner: **(A)** forward and back separately; **(B)** up and down separately; **(C)** forward and back together; **(D)** up and down together; **(E)** spin together.

The visual stimuli were videotaped dynamic scenes of a toy with electric-powered moving joints. The toy was built using the Lego Mindstorms© robot building set and covered with purple felt. The toy was composed of a square “torso” and cylinder “arms.” Five different arm motions were filmed, each in a different video clip (See Figure 2). Four of the motions were considered to comprise a category. These motions might be described as “flapping” or “swinging.” In each of these four motions, the arms moved back and forth in a single plane, as if on hinge joints. In one of the four motions, the arms moved back and forth in unison. In another, they moved up and down in unison. In the third in-category movement, the arms moved back and forth in alternation, and in the fourth, they moved up and down in alternation. In each of the four in-category motions, the arm movements cycled at a rate of approximately one cycle every 2 s.

The fifth motion was qualitatively different than the four in-category motions and was used as an OOC motion. In this motion, the arms moved in a smooth, circular rotation (as if in ball-and-socket joints) in unison. The OOC motion cycled at a rate of approximately one cycle per second. A norming study with adults confirmed our intuitions about the category structure. Five University students were asked, which motion was unlike the other four. All five selected the “OOC” motion, with a 0.2 chance of picking each one, binomial probability <0.001.

In addition to this adult norming, we initially piloted the stimuli in a simple discrimination task. Infants habituated to a single motion, and then at test saw the familiar and two of the four novel motions (either in or OOC). We piloted the stimuli in silence and in the presence of English labeling. In both auditory conditions, infants showed a looking time preference for the novel stimuli, *p*’s < 0.05. Novelty preferences were shown to all motions, so we can be certain that the infants both could discriminate the stimuli and were sufficiently interested in them, making them suitable objects of study. However, this initial piloting also showed that without including the audio during the test phase, infants would frequently lose interest, and so for the three audio conditions, the same audio was played both during habituation and test.

Four audio tracks were created to pair with the video stimuli to create four sets of audio–visual stimuli. Two of these contained language tracks (English and Hebrew), and two were non-language (music and silence). The English language auditory track consisted of a female voice repeating in child-directed speech a pair of labeling phrases, using a nonsense label, “Look, *goppen*. See, *goppen*.” These frames were chosen to be neutral with respect to grammatical category. Six different tokens of this phrase were laid on a single auditory track. Tokens ranged in length from 1.8 to 2.5 s, with a silent pause ranging from 1.2 to 1.75 s between tokens. The five motions were each combined with the auditory track to create five labeling events. Each stimulus pairing was looped such that each event lasted 20 s.

A Hebrew “labeling” phrase was also created to match the structure of the English phrase in all ways, length of tokens, length of pause, and so on. The phrase also matched the English phrase in that it had an alternating “carrier” phrase and repeating “label.” The phrase is translated into English as “Where is the cat? Here is the cat.” The Hebrew translations of “Where is” and “Here is” acted as the alternating carrier phrases while “the cat” (in Hebrew) acted as the consistent label. Given the lack of exposure to Hebrew speech by any of our participants, using nonce Hebrew words was unnecessary. The phrase was spoken by a native Hebrew speaker, a mother of two, in child-directed speech.

The musical track was a short sample of Australian didgeridoo music that contained dynamic pitch and volume elements roughly mimicking the labeling track. Unlike the labeling track, the musical track contained no extended silent portions, just some brief pauses between “notes.” The didgeridoo was chosen because it was likely to be novel, but has a relatively voice-like timbre for a musical instrument. As with both speech conditions, each of the motions was combined with the music and silent audio track to create sets of five events. That is, the same audio accompanied all five stimuli (four within category, one out of category) throughout any particular audio condition, respectively.

#### Apparatus

Infants were tested in a dimly lit experimental room with a color computer monitor (23 cm diagonal) and audio speaker. A Sony brand 8-mm video camera was positioned below the computer monitor. This camera was attached to a Panasonic brand TV in a neighboring room where the experimenter observed the infant. The experimenter used a Macintosh G3, and specially designed software Habit 2000 (Cohen et al., 2000) to present stimuli, record infants’ looking time, and to determine if the infant reached the habituation criterion.

#### Procedure

Infants were randomly assigned to one of four conditions. Infants in the silent condition viewed the visual stimuli in silence. Infants in the English, Hebrew, or music conditions heard their conditions audio during visual presentation throughout the habituation and test phases of the experiment.

Infants sat in their parents’ lap during the procedure, approximately 40 cm from the video monitor. Each trial began with an attention getting video of a green looming circle, accompanied by a bell sound. When the infant looked toward the monitor, the experimenter began a trial by depressing one computer key, and recorded looking time by depressing another key. During each trial, a video clip of a single motion event played until the infant looked away from the computer monitor for more than 0.5 s or until the 20-s trial ended. Infants viewed repeated trials of the same motion event until looking time (averaged across a sliding window of three consecutive trials) decreased 50% from the looking time of the first three trials, or until 20 habituation trials were presented. Infants were recorded, and a random 30% of infants’ videos (from Experiments 1 and 2) were independently coded by a second individual. The two raters agreed on over 80% on what constituted the infant looking away from the screen.

During the habituation phase, infants viewed sequential trials of three in-category motions paired consistently with one of the four audio options (“Look goppen” and “see goppen” in the English condition, and the Hebrew words for “Where is the cat” and “Here is the cat,” in the Hebrew condition, repeated sequences of didgeridoo music for those in the music condition, and silence for those in the silent condition). The fourth in-category motion was used during the test phase. Counterbalancing determined which of the four in-category motions served as habituation events and which served as test events for a given infant.

Habituation was followed by a test phase that contained four trials, presented one at a time in sequence. The first test trial displayed a familiar motion from habituation (i.e., one they already saw), which allowed for a test for spurious habituation (four infants were shown to be spurious habituators, and were removed from analyses; Cohen and Menten, 1981; this first trial was then not included in further analyses). The second and third test trial contained a familiar motion (i.e., one they had already seen) and a novel in-category motion (i.e., the only one of the four they had yet to see). The order of these two stimulus types was counterbalanced across participants. These two trials were used to test for categorization. The fourth test trial displayed the OOC motion. This trial was always last because this was expected to be dishabituating for all infants capable of showing a novelty preference.

### Results

Looking time data were skewed, so a natural log transform was performed prior to analysis.

#### Habituation Phase

Mean looking times for the first and last three trials, and total looking times during habituation are presented in Table 1. A one-way ANOVA revealed no differences in total time during habituation, *F* < 1, *p* > 0.4.

**Table 1:** Mean looking times in seconds (and standard deviations) for habituation in Experiment 1.

Condition	First Three Trials	Last Three Trials	Total Habituation
Silent	10.52 (5.43)	5.46 (1.98)	101.15 (58.91)
Music	12.77 (3.93)	5.31 (1.84)	104.88 (70.47)
English	14.13 (3.95)	5.99 (1.88)	108.66 (68.44)
Hebrew	13.23 (3.91)	5.48 (1.79)	108.66 (54.45)


#### Test Phase Analysis

**FIGURE 3:**
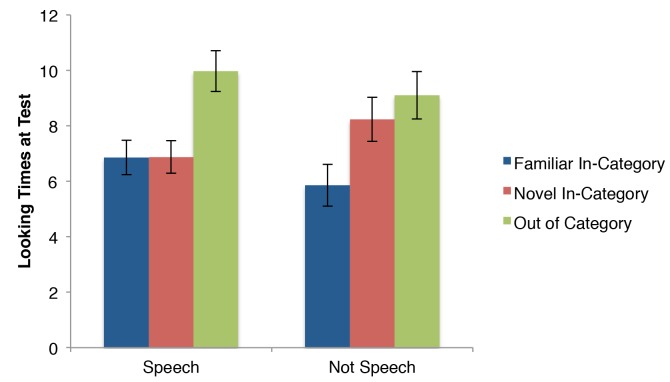
Looking times in seconds (means and standard errors) of infants during the test phase of Experiment 1.

Our primary prediction is that infants who heard speech while the motion stimuli were presented would categorize the stimuli, while infants who either heard nothing or music would not categorize the stimuli. First consider the pattern of results visually, before the statistical analysis. Figure 3 presents the results across the three crucial test trials (again, the first test trial’s purpose is to detect false habituators and is not included in these analyses). Quite similar to the idealized pattern in Figure 1, the Speech conditions (*n* = 54) appear to elicit categorization of the motion stimuli, as familiar and novel in-category stimuli have similarly low looking times. On the other hand, the not speech conditions (*n* = 52) resemble the pattern from Figure 1 showing a lack of categorization because the novel in-category stimulus is closer to the out of category stimulus, than the familiar.

A 2(speech vs. non-speech) × 3(familiar vs. novel vs. OOC) mixed-measures ANOVA revealed the contrast between the two patterns across test trials was statistically significant. There was no main effect of speech, *F*(1,104) = 1.54, *p* = 0.22, $\eta_{p}^{2}$ = 0.015. There was a main effect of trial, *F*(2,208) = 15.18, *p* < 0.0001, $\eta_{p}^{2}$ = 0.126. There were differences between all three trials, with the OOC eliciting longer looking time than the novel in-category stimulus [*t*(105) = 2.66, *p* < 0.01; *d* = 0.25] and the familiar in-category [*t*(105) = 5.2, *p* < 0.0001; *d* = 0.50], and the novel in-category stimulus was significantly different from the familiar stimulus [*t*(105) = 2.89, *p* < 0.01; *d* = 0.29]. Most importantly, there was a significant speech × trial interaction, *F*(2,208) = 3.47, *p* < 0.05, $\eta_{p}^{2}$ = 0.032 consistent with the prediction that the infants categorized the stimuli when exposed to speech, but not otherwise.

**FIGURE 4:**
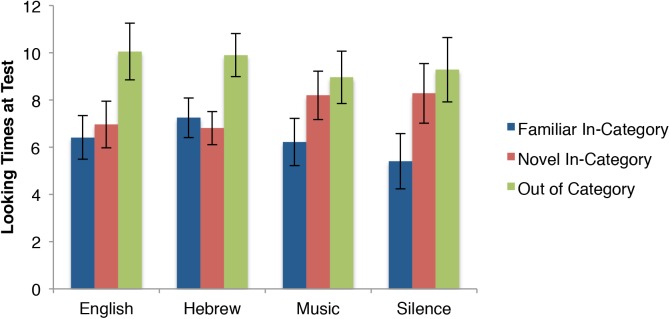
Looking times in seconds (means and standard errors) of infants during the test phase of Experiment 1 for each of the four audio conditions.

Next, consider the pattern across test trials for all four conditions separately in Figure 4. The two speech conditions’ patterns are quite similar to each other, as are the two non-speech conditions to each other (respectively).

Both the “speech promotes categorization” and auditory overshadowing accounts would expect relatively little differences between English and Hebrew conditions (with the exception that the unfamiliar Hebrew speech may elicit even more overshadowing). The 2(English vs. Hebrew) × 3(familiar vs. novel vs. OOC) mixed-measure ANOVA revealed a pattern consistent with both accounts. There were 25 participants in the English condition and 29 participants in the Hebrew condition. There was no main effect of language, *F*(1,52) = 0.32, *p* = 0.57, $\eta_{p}^{2}$ = 0.006, nor a language × trial interaction, *F*(2,104) = 0.03, *p* = 0.97, $\eta_{p}^{2}$ = 0.001. There was a main effect of Trial, *F*(2,104) = 8.63, *p* < 0.001, $\eta_{p}^{2}$ = 0.143. Infants looked significantly longer at the out of category stimulus than they did to the familiar stimulus [*t*(53) = 3.79, *p* < 0.01; *d* = 0.51]. They also looked significantly longer at the out of category stimulus than they did at the novel, within category stimulus [*t*(53) = 3.36, *p* < 0.01; *d* = 0.45]. There was no difference in looking time to the familiar and novel, within category stimuli [*t*(53) = 0.29, *p* = 0.77, *d* = 0.04]. This pattern reflects categorization of the stimuli.

Unlike with comparing the two speech conditions, the “speech promotes categorization” account and the auditory overshadowing account make quite different predictions when comparing the two non-speech conditions. The former predicts that both non-speech audio conditions would elicit novelty preferences to the novel in-category stimulus, while the auditory overshadowing account predicts that music should reduce discrimination between the familiar and novel, thus showing no such novelty preference. A 2(silent vs. music) × 3(familiar vs. novel vs. OOC) mixed-measures ANOVA revealed a pattern more consistent with the “speech promotes categorization” account. There were 29 participants in the music condition and 23 in the silent condition. There was no main effect of Audio, *f*(1,50) = 0.37, *p* = 0.61, $\eta_{p}^{2}$ = 0.005, nor an audio × trial interaction, *f*(2,100) = 0.50, *p* = 0.61, $\eta_{p}^{2}$ = 0.010. There was a main effect of trial, *f*(2,100) = 9.42, *p* < 0.001, $\eta_{p}^{2}$ = 0.158. Infants looked significantly shorter at the familiar stimulus than at both the out of category stimulus [*t*(51) = 3.51, *p* < 0.01; *d* = 0.50] and the novel in-category stimulus [*t*(51) = 3.86, *p* < 0.01; *d* = 0.54]. There was no difference between the two novel stimuli [*t*(51) = 0.45, *p* = 0.65; *d* = 0.06]. This suggests the infants did not categorize the stimuli, but did discriminate between familiar and novel.

In sum, for the two speech conditions the novel in-category motion patterned with the familiar motions and not with the OOC motion (suggesting categorization), while for the two non-speech conditions, the novel in-category patterned with the OOC motion and not with the familiar in-category motion (suggesting the infants did not categorize).

### Discussion

In this experiment, 9-month-old infants were habituated to a set of novel movements. After habituation, and consistent with our hypothesis, infants treated a novel in-category exemplar as novel (like the OOC exemplar and unlike the other within category stimuli) in silence and when listening to didgeridoo music. Infants behaved differently in the test phase if they had listened to either English or Hebrew speech during the habituation phase. In those conditions, the infants treated the novel in-category stimulus as familiar (like other in-category stimuli, and unlike the OOC stimulus).

We believe these findings are best explained by a facilitative influence of labeling speech on visual categorization. However, there are two alternative explanations. One is that somehow the speech conditions produced more false habituators or fatigued infants, that is, these infants were incapable of showing a novelty preference. However, we ruled out that explanation out with the final test trial. All conditions showed a novelty preference to this OOC stimulus.

The other alternative explanation is that attending to the speech overshadowed the infants’ visual processing, reducing within category discrimination (Robinson and Sloutsky, 2004, 2007). We believe this is unlikely because if overshadowing were the cause, then the music condition should also have shown no novelty preference within the category, as unfamiliar sounds should be even more resource demanding (Robinson and Sloutsky, 2007; Sloutsky and Robinson, 2008). In addition, we piloted the visual stimuli in the presence of our English stimuli, and motions were discriminated (see above). However, to completely rule out auditory overshadowing as an explanation of the current pattern, we now present a discrimination study identical in method to Experiment 1 except infants habituated to only a single motion, and only the two speech conditions were used. Habituating to a single exemplar should not be enough to form a category (see Cohen and Strauss, 1979) and so if infants can discriminate the motion stimuli while hearing speech, then their pattern across the test trials should resemble the non-speech conditions of Experiment 1 wherein they looked longer at the novel in-category stimulus than the familiar.

## Experiment 2

### Methods

This study was approved by the Institutional Review Board of the University of Texas at Austin. We obtained written informed consent from the participants’ parents or legal guardians in line with the study approval.

#### Participants

Fifty-two 9-month-olds (±2 weeks; *M* = 272 days; SD = 9.2 days) participated (27 boys and 25 girls). Of these, two were eliminated for fussiness, two were eliminated for parent interference, five were eliminated for computer error, one was eliminated for not having reached the gestational age of 33 weeks (no other infant was below the cutoff of 36 weeks), and three were eliminated because there was no sign of habituation, having attended 20 consecutive trials without reaching criterion. Two were eliminated for maximum looking time on the first test trial. This left 37 infants contributing to the data analysis.

#### Stimuli and Apparatus

These were the same as the speech conditions for Experiment 1.

#### Procedure

The infants habituated to a single motion event accompanied by either English or Hebrew labeling speech. The test phase was structured as in Experiment 1, with four test trials. The first was the familiar motion, and the last was the OOC motion. The middle two were one of the three novel in-category motions, and the other was the familiar motion again. The order of these two was counterbalanced across participants. All possible comparisons of two in-category motions were covered across participants, just as they were in Experiment 1, meaning that any differences across studies must be due to how the infants processed the stimuli during habituation.

### Results

#### Habituation

Mean looking times for the first and last three trials, and total looking times during habituation are presented in Table 2. T-tests revealed no differences in habituation time. As in Experiment 1, all analyses were conducted using log-transformed looking time data.

**Table 2:** Mean looking times in seconds (and standard deviations) for habituation in Experiment 2.

Condition	First Three Trials	Last Three Trials	Total Habituation
English	13.54 (4.97)	6.01 (2.17)	117.97 (63.31)
Hebrew	12.91 (3.25)	5.98 (1.67)	110.78 (45.37)


#### Discrimination Analysis

**FIGURE 5:**
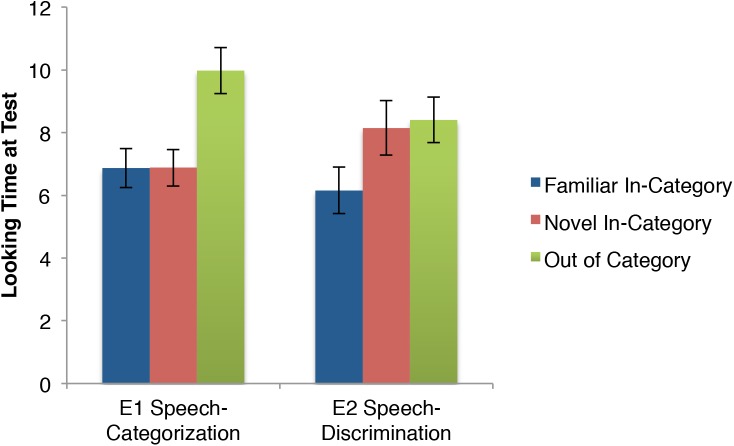
Comparing looking time at test (in seconds; means and standard errors) between the Experiment 1 and Experiment 2 speech conditions.

First consider the pattern of both speech conditions together and compare them to the pattern of both speech conditions from Experiment 1, presented again here for ease of comparison in Figure 5. These infants across the two experiments heard identical audio and were shown identical visual stimuli during the test phase. The only difference was whether they habituated to three stimuli (E1) or one (E2). Figure 5 shows distinct patterns across the two experiments, with now both novel stimuli eliciting similar looking times, both apparently longer than the familiar trial.

**FIGURE 6:**
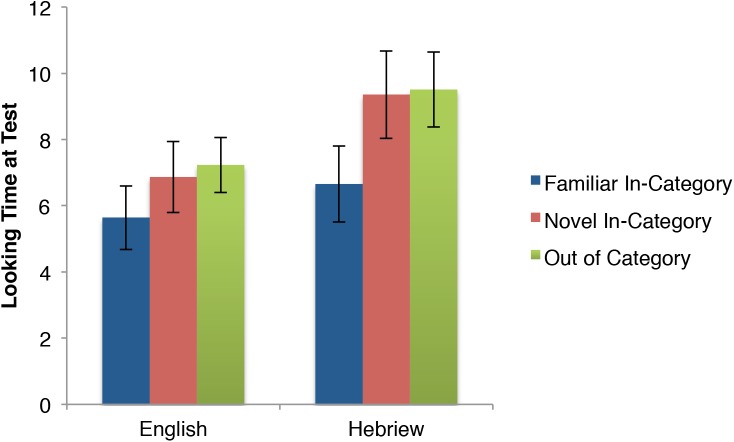
Looking times (in seconds; means and standard errors) during the test phase of Experiment 2.

Next consider the two audio conditions of E2 independently in Figure 6. Both show the novel in-category stimulus as eliciting looking times closer to the novel OOC stimulus than the familiar (though granted this pattern is more visually clear in the Hebrew condition).

To test for discrimination, we conducted a 2(English vs. Hebrew) × 3 (familiar, novel in-category, OOC) mixed-measures ANOVA. There were 18 participants in the English condition and 19 in the Hebrew condition. Consistent with Experiment 1, the two speech conditions did not differ from each other reliably. There was no main effect of audio condition as the overall longer looking times in the Hebrew condition did not reach significance, *F*(1,35) = 2.75, *p* = 0.11, $\eta_{p}^{2}$ = 0.073. More importantly, there was no audio condition × trial interaction, *F*(2,70) = 0.33, *p* = 0.72, $\eta_{p}^{2}$ = 0.010, but there was a main effect of Trial, *F*(2,70) = 6.71, *p* < 0.01, $\eta_{p}^{2}$ = 0.164. That is, both audio conditions elicited the same pattern across the test trials consistent with discrimination.

Infants looked significantly longer to the novel, within category trial than to the familiar trial [*t*(36) = 2.54, *p* < 0.05; *d* = 0.42]. They also looked longer to the out of category stimulus than to the familiar stimulus [*t*(36) = 3.56, *p* < 0.01; *d* = 0.64]. There was no difference in looking time to the novel within category stimulus and the out of category stimulus [*t*(36) = 0.09, *p* = 0.38; *d* = 0.15]. Unlike in Experiment 1, where the novel in-category motion patterned with the familiar for the speech conditions, showing categorization, here it patterned with the novel OOC motion, demonstrating discrimination.

## General Discussion

Two experiments showed that native and non-native speech facilitated the categorization of motions, without reducing discrimination. These findings are first to show the categorization of novel stimuli in the presence of speech that in silence showed little evidence of categorization. These findings join a now substantial literature on the importance of language in categorization in infancy and across the lifespan (e.g., Namy and Waxman, 2000; Bowerman and Choi, 2001; Gentner, 2003; Yamauchi, 2005; Lupyan et al., 2007; Ferry et al., 2010; just to name a few).

We had three research questions: (1) Can labeling speech enable categorization of visual stimuli when the same stimuli are not categorized in silence? (2) Will other auditory stimuli have the same effect? (3) Does the effect of speech extend to motion categories? We will now discuss our results, their theoretical implications, and topics for future research for each question.

### Can Labeling Speech Facilitate Visual Categorization?

Using the generalization of habituation paradigm, we show that the same set of exemplars from an artificial motion category are categorized together (E1) and discriminated from each other (E2) in the presence of speech, while they are only discriminated from each other in silence (E1). This result is in line with the theoretical perspective of Balaban and Waxman (1997). However, we expand upon their studies in several ways. One is that our category is artificial, enabling us to claim our speech conditions enabled learning of the category, and did not just cue a known category in memory, as could be argued for Balaban and Waxman’s results (Plunkett et al., 2008). Second, we present an independent test of discrimination, essentially ruling out auditory overshadowing of visual processing as an explanation for the current effects (see Robinson and Sloutsky, 2004, 2007). Third, we use a stimulus set that was not categorized in silence (or in the presence of non-speech audio). This is crucial because one criticism of Waxman and colleagues’ account is that categorization does not need labels; children form them well enough on their own (Robinson and Sloutsky, 2007). Finally, our measure of categorization, generalization from familiar to novel in-category members, in concert with the independent test of discrimination is a more direct operationalization of the definition of categorization, which is the treatment of discriminable stimuli as an equivalence class (e.g., Younger and Cohen, 1983). Even though we improved on the typical measure, our analyses further include the comparison typically used in the literature, a novelty preference for an OOC stimulus over a novel in-category member.

Because we use a different measure of categorization than this literature does typically, it is worth considering whether our interpretation would change if we did rely on the typical measure. Perhaps the typical measure (preference for an OOC stimulus to a novel in-category stimulus) is a less direct measure of categorization, but still provides a form of evidence for it. Although, this may be true, the pattern considering this other measure does not suggest a different interpretation of the present results because infants in the silent and music conditions showed lower looking times for the familiar stimulus compared to both kinds of novel stimuli, and showed equally long looking times to both kinds of novel stimuli. On the other hand, infants in the two speech condition showed equally longer looking times to the OOC stimulus than both the familiar and novel in-category exemplars.

As our findings across the board are quite a different pattern than Robinson and Sloutsky (2007) predictions, they warrant further discussion. First of all, we should make clear that although we do not think auditory overshadowing is a viable explanation of the current results, we certainly are not arguing that it is not a real phenomenon or that it fails to explain other experimental findings. It is worth contrasting our methods to consider whether the differences will lead to insight into the nature of auditory overshadowing.

Using three separate test conditions with the same stimuli, those authors found that 8-month-old infants were most likely to categorize (prefer to look at a novel OOC exemplar over a novel in-category exemplar) in a silent condition and least likely to categorize in an unfamiliar noise condition. Categorization performance for infants listening to labels was somewhat in the middle and, as in the other acoustic condition, preference for OOC novelty was not above what would be expected by chance. Robinson and Sloutsky included the silent condition, arguing that this is a proper control for testing effects of acoustic stimuli on categorization performance. We agree with this approach, but some aspects of the Robinson and Sloutsky design prohibit them from taking full advantage of this control condition. The use of a familiarization phase instead of habituation to criterion, in particular, prohibits the researchers from taking into account an increase in stimulus complexity that comes with the addition of acoustic stimuli. Increasing the complexity of a given stimulus set will cause infants to take a longer time to process those stimuli (Hunter and Ames, 1988). If given only a limited amount of time to process a stimulus set as a category, as in the familiarization paradigm, adding additional components to a stimulus set might mean that infants no longer have enough time to fully process the stimuli. This would be true for additional complexity in any modality, including additional acoustic stimuli. Robinson and Sloutsky provide no evidence that (as their auditory overshadowing hypothesis posits) the acoustic nature of their stimuli affects categorization in a manner different than would additional visual complexity. The task of balancing complexity across modalities seems prohibitive. Rather than trying to balance judgments of complexity across sensory modalities to keep processing loads constant in a familiarization paradigm, we suggest the method of habituation to criterion, as we used here. This method, we argue, allows each individual participant to achieve his or her own required processing time, and is a better method for equating across stimulus sets of varying complexity.

Additionally, our visual stimuli were different from Robinson and Sloutsky’s. We used a novel motion category, while they used a familiar object category. Perhaps the novelty of our stimuli made them more interesting and so visual processing was not overshadowed to the same degree. The same could be said for our moving versus their still stimuli. However, both of these suggestions need further testing and direct comparisons.

Moreover, as discussed above, we used a different categorization measure than Robinson and Sloutsky (2007). Like Balaban and Waxman (1997), their measure differs from ours not only in that it compares novel OOC stimuli to novel in-category stimuli, but the two are presented simultaneously, while the generalization of habituation paradigm presents stimuli at test sequentially. Future research should directly compare how these measures pattern to determine to what degree they are measuring the same process (see Oakes and Ribar, 2005 for discussion of this issue during familiarization).

### How Important Is It That the Auditory Stimulus Is Labeling Speech?

Our second goal was to ask to what degree these effects were unique to labeling speech that contained familiar speech sounds. Both Hebrew and English speech produced the same effects. Both audio tracks were structured similarly, with alternating carrier phrases and repeating labels. That Hebrew produced the same effects suggests that the familiarity of the carrier phrase is not at the effect’s impetus. This leads one to ask, to what degree does it matter that the speech was labeling at all? One may think it does not, as infants at this age seem not to directly assign labels to categories in laboratory settings (and that perhaps words act more as features of category exemplars rather than as a referential label for the category as a whole until infants are 12 months old, see Gliozzi et al., 2009). Thus it is not clear whether infants are interpreting the English speech, let alone the Hebrew, as labeling. However, other findings suggest it does matter, as two labels may produce different effects than one (Waxman and Braun, 2005; Plunkett et al., 2008, though this is also consistent with the above word-as-feature account), and affective non-labeling speech (e.g., “ooh” or “yuk”) does not play the same role in individuating objects as labels do (Xu, 2002). Disentangling the role of labeling from other forms of speech is an important topic of future research.

Beyond speech, we asked if complex voice-like music could enable categorization. We found no evidence for that possibility, as infants in the music condition showed a in-category novelty preference. These data are in support of the findings of Waxman and colleagues who have provided evidence for a facilitative effect unique to language using experimental paradigms that differ from ours (Balaban and Waxman, 1997; Fulkerson and Waxman, 2007). Our findings are not consistent with those reported by Roberts and Jacob (1991). Those authors report a facilitative effect of music on categorization. It has been suggested that the effect may stem from auditory stimuli that are suitably complex. The music we used in our study, however, was also complex, but was also unfamiliar, from an unfamiliar culture and instrument. The infants in our study were also younger (9 months) than those in the studies reported by Roberts and Jacob (15 months). It may be that the facilitation effect is a function of the age of the participant, and the complexity and familiarity of the stimuli. Clarification of the discrepancy would require further research.

### Does Labeling Speech Only Affect Object Categorization?

By and large, previous work on the question of labels affecting visual categorization has focused on object categorization. Some have theorized that labels primarily direct attention to whole objects (Baldwin and Markman, 1989), so it is worth asking if these effects extend to motion categories. We found that speech does enable motion categorization. However, we recommend caution in interpreting these findings. Words refer to categories, thus much of the impetus for research on labels and categorization is to investigate the cognitive prerequisites of word learning. Gentner (1982) argues that verbs are learned later than nouns because mapping words to the relational representations of action and motion events is more difficult than mapping words to the “perceptually pre-parsed” featural representations of objects. In a simple parallel, one might note that nouns often refer to object categories and verbs often refer to motion categories, and that in our study, labels facilitated categorization of motion with infants of the same age as those for which object categorization has been facilitated (Balaban and Waxman, 1997). With this simple parallel, it is tempting to make the leap that 9-month-olds are equally ready to learn verbs as they are to learn nouns, potentially contradicting Gentner (1982) account.

We do not advocate making such a theoretical leap, however. Categorizing motions is one of many potential prerequisites for verb learning. Verbs are predicates that bind arguments forming relationally complex event structures (Gentner, 1982). Verbs do not just refer to motion categories; they often refer to causal-event categories. In addition, children must learn which aspects of motion their native language’s verbs encode (which has great cross-linguistic variation, see e.g., Talmy, 1975). The results of the study here are not applicable to a great portion of the complexity of verb learning. Future research will investigate the ability for infants to form representations of causal events, and other kinds of relational categories (see Gentner et al., 2011; Ferry et al., 2015; Yin and Csibra, 2015 for work with infants and young children, and Goldwater et al., 2011; among others for related work with adults), and how labeling may interact with this process.

Our findings do illustrate a facilitative effect of language on categorization. That the motions were categorized only in the presence of labeling speech, and by infants of such a young age, suggests that infants who are preparing to enter into word learning possess some cognitive mechanism that uniquely relates language with the categories in the infants’ environment to which language refers. We cannot yet say whether the relationship is a direct one, in which infants make some attempt to relate the forms of language to categories in the environment, or if it may be indirect, in which language perhaps simply raises attention to what is consistent across labeling events, or perhaps that a label simply acts as salient feature shared by the category’s exemplars. Our findings do suggest that this effect is not limited to object categories that are defined by static visual properties such as shape and color. Rather, this relationship between language and the organization of information in infants’ environment may be general, such that motion and, possibly, other consistent elements may serve as organizers. Young infants are faced with a daunting task of organizing a wide variety of environmental stimuli into category structures within different ontological domains, including objects, motions, spatial relationship, and qualitative properties. Language may serve as one of many facilitators in that process.

## Author Contributions

All three authors designed the materials, procedure, and worked on the manuscript. MG and RB collected and analyzed the data.

## Conflict of Interest Statement

The authors declare that the research was conducted in the absence of any commercial or financial relationships that could be construed as a potential conflict of interest.
